# Independent Heritability of Aversive Learning Influences on Cocaine-Seeking and Punishment Resistance in Rats

**DOI:** 10.1523/ENEURO.0083-25.2026

**Published:** 2026-06-19

**Authors:** Maya Eid, Dominika Pullmann, Paul J. Nietert, Rachel Lipat, Courtney Rowley, Thomas C. Jhou

**Affiliations:** ^1^ Department of Neurology, Icahn School of Medicine, New York, New York 10029; ^2^ Department of Neurosciences, Medical University of South Carolina, Charleston, South Carolina 29425; ^3^ Department of Plastic Surgery, NYU Grossman School of Medicine, New York, New York 10016; ^4^ Department of Public Health Sciences, Medical University of South Carolina, Charleston, South Carolina 29425; ^5^ Department of Neurobiology, University of Maryland School of Medicine, Baltimore, Maryland 21201

**Keywords:** aversion, cocaine, heritability, heterogeneous stock, punishment

## Abstract

Cocaine produces widely recognized rewarding effects but also produces aversive effects that occur several minutes after rewarding effects dissipate. Prior work shows these aversive effects are particularly strong in some animals, in whom they produce conditioned avoidance effects that reduce overall cocaine-seeking. The sources of these individual variations are largely unknown, but we found evidence for contributions from heritable influences. Using outbred male and female Sprague Dawley and heterogeneous stock rats, we found that offspring of individuals expressing higher versus lower levels of conditioned avoidance to cocaine on a runway operant task are themselves higher or lower in this trait. These results did not differ between sexes and are consistent with a heritable influence driving conditioned avoidance of cocaine, which could either be genetic or nongenetic. Runway latency to obtain cocaine also differed markedly between seven inbred rat strains (tested in males only), again consistent with a heritable influence. Several control tasks showed that variations in cocaine avoidance were not explained by differences in exploratory locomotion, overall motivation, or resistance of food-seeking to footshock punishment. Notably, the latter punishment resistance task has been linked to addiction-like behaviors in its own right, and performance on this task also varied considerably between inbred strains but did so independently of cocaine avoidance. Hence, punishment resistance and cocaine avoidance may be influenced by independent heritable factors.

## Significance Statement

Cocaine's strong rewarding effects critically drive drug intake, but this intake can be reduced by cocaine's aversive effects, which occur a few minutes after rewarding effects dissipate and whose intensity varies considerably between animals. Using inbred rat strains and selective breeding, we show that conditioned avoidance to cocaine may have a substantial heritable component, which could be either genetic or nongenetic. We also show that aversive effects of cocaine vary independently of other aversive tasks, including resistance to punishment.

## Introduction

Cocaine produces strong rewarding effects that drive its intake in humans and animals, but this drug also produces an aversive “crash” occurring 15–30 min after acute drug exposure, shortly after cocaine's acute rewarding effects have dissipated (Ettenberg et al., 1999; Ettenberg, 2004). Both rewarding and aversive effects can be measured using standard conditioned place preference (CPP) task, as animals show a preference for a cocaine-paired chamber if placed into the conditioning chamber immediately after receiving cocaine, while many animals will instead avoid the cocaine-paired chamber if placed into conditioning chambers 15 min after receiving cocaine (Ettenberg et al., 1999). Prior studies have shown that the magnitude of this aversive effect varies markedly between individual rats and influences not only CPP but also several operant tasks (Ettenberg et al., 2015; Parrilla-Carrero et al., 2021; Chao et al., 2023), including lever press operant cocaine-seeking tasks, as well as an operant runway task in which animals must traverse a 5 ft corridor to obtain cocaine (Geist and Ettenberg, 1997; Ettenberg et al., 1999, 2015; Ettenberg, 2004; Maroteaux and Mameli, 2012; Jhou et al., 2013; Parrilla-Carrero et al., 2021; Chao et al., 2023). Hence, a better understanding of cocaine's aversive effects and their individual differences might help explain why some animals seek cocaine more than others.

Prior studies have identified several brain circuits that could drive cocaine's delayed aversive effects, including the amygdala, extended amygdala, entopeduncular nucleus (EPN), lateral habenula (LHb), and rostromedial tegmental nucleus (RMTg; Wenzel et al., 2011; Shelton et al., 2016; Klein et al., 2019; Li et al., 2020). However, it is not known why these circuits might drive stronger avoidance in some animals than others. One possible substrate of these individual differences is the RMTg, which drives punishment learning and shows greater cocaine-induced activity in animals with high levels of cocaine avoidance (Parrilla-Carrero et al., 2021), whereas other areas, such as LHb and EPN, did not show such differences. Hence, synaptic signaling differences in RMTg neurons might explain individual differences in avoidance behavior (Parrilla-Carrero et al., 2021; Chao et al., 2023), but it is still unknown what might cause these cellular mechanisms to differ.

Similarly to cocaine, other misused drugs, such as alcohol and nicotine, also produce aversive effects that influence drug-seeking, and in the case of alcohol and nicotine, such effects may be partly influenced by heritable genetic factors (Fowler and Kenny, 2014; Parker et al., 2020). These findings led us to examine whether cocaine avoidance behavior might also be under heritable influences, which would pave the way for more extensive studies examining the molecular basis of such heritability. Toward these ends, we conducted heritability tests using Sprague Dawley (SD) rats, as well as a population of heterogeneous stock (HS) rats that are composed of a mixture of eight inbred strains and that were developed to facilitate studies of heritable complex traits (Hansen and Spuhler, 1984; Solberg Woods and Palmer, 2019). We screened rats on a runway operant task, then selected high-avoider and low-avoider pairs for breeding, and found that offspring of these breeders exhibited correspondingly higher versus lower latencies to obtain cocaine. We also examined several different inbred rat strains and found marked differences between strains in cocaine avoidance. Both findings are consistent with a heritable influence for this trait. Finally, we used several control tasks to show that differences in cocaine-seeking were not readily explained by nonspecific differences in motivated behavior, exploratory locomotion, or aversive learning.

## Materials and Methods

### Animals

We initially obtained 48 SD rats (males and females) from Charles River Laboratories and 16 HS rats from a breeding colony at the Medical College of Wisconsin (overseen by Dr. Leah Solberg Woods, now at Wake Forest School of Medicine). From these vendor-purchased rats, we bred an additional 107 SD rats over two generations of selective breeding and an additional 33 HS rats over a single generation.

Five female SD rats were excluded due to catheters failing patency testing at the end of runway behavioral testing, and three were excluded due to complications from surgery. Six HS rats were excluded due to catheters failing patency at end of testing.

Some rats were excluded due to failure to show exploratory behavior on the runway apparatus, as defined by failing to enter the goal box within 60 s of door opening on four consecutive habituation sessions. These included one male SD rat and several inbred rats noted below.

We also used the following inbred strains: Agouti (ACI; *n* = 16), Brown Norway (BN; *n* = 11, plus four excluded due to runway habituation failure), Buffalo (BUF; *n* = 8), Fischer (FSH; *n* = 12), Lewis (LEW, *n* = 9), M520 (*n* = 14, plus two excluded due to runway habituation failure and two excluded due to catheter patency failure), and Wistar Kyoto (WKY; *n* = 18, plus one excluded due to runway habituation failure). ACI, BN, and FSH rats were purchased from Harlan Laboratories; BUF, LEW, and WKY rats were from Charles River Laboratories.

All rats weighed 200–350 g upon delivery from vendor and were individually housed in standard ventilated cages in a vivarium maintained at 22°C and with a 12/12 h light/dark cycle (lights on at 6:00 A.M.), with food and water provided *ad libitum*, unless otherwise stated. Procedures conformed to the National Institutes of Health Guide for the Care and Use of Laboratory Animals and institutional protocols approved by our university's Institutional Animal Care and Use Committee. All animals were tested during the light phase.

### Surgery

Rats were anesthetized with isoflurane and fitted with indwelling intravenous catheters inserted into the right jugular vein and subcutaneously passed to a guide cannula exiting the animal's back. After surgery, catheter patency was maintained via daily flushing with 0.05 ml of taurolidine-citrate solution. Animals recovered for 7 d prior to behavioral testing. Catheter patency was assessed after the final cocaine test, through observation of the loss of the righting reflex after intravenous injection of methohexital (Brevital, 2.0 mg/kg in 0.1 ml) or propofol (5–10 mg/kg in 0.1 ml). Rats that failed to lose the righting reflex were removed from the study.

### Behavioral testing

#### Runway operant cocaine-seeking

Methods are similar to our previous work (Jhou et al., 2013). Briefly, the runway consists of two opaque plastic compartments (“start” and “goal,” 25 × 10 × 17 cm length/width/height) connected by a 170-cm-long corridor with manually operated doors between the start/goal boxes and corridor (Med-Associates). On test days, rats were tethered to an intravenous line and then placed into the start box for 30 s, after which doors were opened to allow free exploration of the apparatus. After entry into the goal box, the experimenter closed the goal compartment doors, and the computer activated a syringe pump to deliver cocaine (0.75 mg/kg, i.v.) over a roughly 10 s period. Dose was calculated individually for each animal, based on weights taken each day prior to the experiment, and entered into a Med-PC software program that calculated the appropriate syringe pump rotation duration. After cocaine infusion, rats remained in the goal compartment for 5 min before being returned to their home cage. A recorded a “timeout” if animals failed to enter the goal compartment after 20 min (SD rats) or 15 min (HS rats). Upon reaching the timeout, animals were gently nudged by the experimenter into the goal box, given intravenous cocaine, and retained there for 5 min, ensuring that all animals received the same exposure to both cocaine and the goal compartment.

Prior to cocaine trials, animals received habituation trials in which they freely explored the entire apparatus without cocaine. Rats received a minimum of two habituation trials, with additional trials until two consecutive habituation trials produced latencies <60 s. Animals that failed to enter the goal box within 60 s for four consecutive sessions were excluded from further study. No animal required >8 habituation sessions to either be designed as habituated or excluded. Animals then received seven cocaine trials, at most twice daily and at least 4 h apart, during the light portion of the 24 h diurnal lighting cycle, over a period of 4–5 d. In all trials, we tabulated both the latency to reach the goal box (after exiting the start box) and the number of run reversals, defined as a change in run direction spanning a minimum of two photobeams (∼12 in).

#### Progressive ratio (PR) and punishment resistance

Training procedures are identical to those we previously described (Vento et al., 2017). Briefly, animals are food restricted to 85% of initial body weight, trained to lever press for food pellets (45 mg, Bioserv, catalog #F0021) on six sessions of a fixed-ratio 1 (FR1) schedule, with 35 trials each session using standard rat operant chambers located inside sound-attenuating cabinets (Med-Associates). Animals met criteria if they obtained all 35 rewards within a 30 min period. All animals met this criteria within 2–5 sessions. After this initial training, animals were then tested either on the PR task with progressively increasing effort requirements (lever presses required per pellet; Richardson and Roberts, 1996) or progressively increasing punishment (i.e., a footshock of increasing intensity presented 1 s after food pellet delivery). In the PR task, the lever ratio started at FR1 and increased after each completed trial using the following sequence of values: 1, 2, 4, 9, 12, 15, 20, 25, 32, 40, 50, 62, 77, 95, 118, 145, 178, 219, 268, 328, 402, 492, 603, 737, 901, 1,102, 1,347, 1,647, 2,012. Starting with the FR20, this represents a 20–25% increase between successive values, forming a roughly geometric progression. In the punishment task, no footshock is delivered on the first five trials, and starting on the sixth trial, footshock is delivered with an intensity of 0.25 mA and duration 0.3 s. The magnitude (but not duration) increases every three trials thereafter on the following schedule of values: 0.25, 0.375, 0.5, 0.75, 1.0, 1.25, 1.5, 2.0, 2.5, 3, 4, 5, 6, and 8 mA. After the 0.375 mA value, the relative increase between successive shocks is 20–33%, making this also an approximately geometric increase. In the PR task, rats time out if 2 min passed without a lever press. In the punishment task, a timeout is defined by three consecutive trials with >30 s passing without a lever press. For both tasks, the breakpoint is the last successfully completed ratio or shock intensity endured before timeout.

#### Locomotion

Exploratory locomotion in a novel environment was recorded in operant chambers (12″ wide, 9.5″ deep, and 11.5″ high) placed inside sound-attenuating cabinets (Med-Associates). Movement within the chamber was detected via interruptions of an array of four photodetector/emitter pairs. Locomotor counts were averaged over two 30 min sessions on consecutive days.

#### CPP/CPA

We used an unbiased design in which the cocaine-paired side was chosen randomly for each animal, independently of preconditioning (baseline) bias. We used a three-chambered apparatus with two conditioning chambers having dark versus light colored walls and different floor textures (dimensions 11″ wide × 8″ deep and 8″ tall), with a third small neutral chamber in the center (5″ wide × 8″ deep and 8″ tall). The dark and light compartments had small incandescent lamps in the ceiling, with the light in the dark-walled chamber set to a higher illumination than the light-walled chamber.

On the first day of this experiment, animals were placed in the central chamber and allowed to freely explore all three chambers for 15 min, thus establishing a baseline preference score. On 4 subsequent days, rats received two conditioning sessions per day in which intravenous saline or cocaine (0.75 mg/kg) were given in the morning or afternoon (counterbalanced between days) in their home cage and then placed in one of the test chambers (saline or cocaine-paired) immediately (for CPP tests) or after remaining in the home cage for a 15 min delay (for CPA tests). One day after final conditioning sessions, animals received test sessions in which rats were placed into the central compartment and freely explored all three compartments for 15 min. Time spent in each compartment was recorded. The final preference score was calculated as the postconditioning preference (time in cocaine-paired chamber minus time in saline-paired chamber) minus the preconditioning preference. Two separate tests (CPP and CPA) were conducted in each rat, in a counterbalanced order, with three extinction sessions between the two tests in which rats explored all chambers without drug. The preference score of the third extinction session was used as the new baseline for the second test.

#### Shuttlebox shock escape

Rats were placed into a shuttlebox having two equal sized compartments and a motorized doorway connecting the two. At 2 min intervals, footshock was delivered via a floor grid, which terminated after the rat crossed through the doorway. Photobeams measured the latency to reach the other side. Ten trials were given for each session, and average latency was recorded for each rat. Shock intensity was fixed during each session, and two sessions were conducted for each rat at each shock intensity. Shocks were tested in an ascending order: 0, 0.15, 0.25, 0.35, 0.5, and 0.7 mA.

#### Lever press self-administration

Some animals were trained on a daily 2 h session of cocaine self-administration during the light cycle, in standard Med-Associates–operant chambers equipped with two levers (one active and the other inactive), a house light, cue light, and tone generator. The designated active lever delivered an infusion of cocaine (0.75 mg/kg/infusion, calculated in the same manner as for the runway task) dissolved in sterile 0.9% saline, along with a compound cue (light + tone) on an FR1 schedule of reinforcement. Following each infusion, a 20 s time out was signaled by the loss of illumination of the house light, during which active lever presses had no programmed consequence. Responses on the inactive lever were recorded but had no programmed consequence. Two days prior to the start of training, rats were food restricted to 15 g of chow per day to facilitate acquisition of SA (Carroll, 1998). All rats received *ad libitum* access to food once SA training was done. After reaching criterion (a minimum of 10 d of self-administration >15 infusions, including the last 3 d, and discrimination between the active and inactive levers), animals were tested for punishment resistance, in which a footshock was delivered 1 s after cocaine infusions, on the same escalating schedule as described for punished food-seeking.

### Histology

Rats were transcardially perfused with saline and 10% formalin; brains were removed and postfixed overnight in 10% formalin, equilibrated in 20% sucrose, sliced into 40 µm sections, and immunostained for c-Fos induced by cocaine (10 mg/kg, i.p.) using rabbit anti-c-Fos (1:10K, EMD Millipore) as described previously (Smith et al., 2019).

### Experimental design

Selective breeding:

1. SD breeding: we tested 48 SD rats on the runway. We classified the animals into high and low avoiders using a median split. For the breeding, we selected four low-avoider pairs and two high-avoider pairs in the top or bottom quartiles of the distribution of run latencies. This selection process was then repeated on the resultant offspring with six pairs from the first generation (three pairs of low-avoiders and three pairs of high-avoiders), yielding a second generation of offspring. The first generation yielded a total of *n* = 11 males and *n* = 10 females of high avoiders and *n* = 29 males and *n* = 20 females of low avoiders. The second generation yielded *n* = 8 males and *n* = 9 females of high avoiders and *n* = 8 males and *n* = 8 females of low avoiders. A subgroup of all offspring tested on runway was used for locomotion testing, and 6 out of the 17 high-avoider second–generation offspring were tested on self-administration.
2. HS breeding: we tested 16 HS rats (eight males, eight females) on the runway task and generated two mating pairs each of the highest and lowest run latencies (four total litters). The breeding yielded a total of *n* = 9 males and *n* = 8 females of high avoiders and *n* = 6 males and *n* = 10 females of low avoiders. A subgroup of all offspring was tested on the runway first, followed by control tasks in the following order: locomotor response in a novel chamber, PR, progressive punishment, elevated plus maze, and CPP/CPA.

In both SD and HS rats, realized heritability (*h*^2^) is calculated from the slope of the regression line fit to a scatterplot of offspring phenotype (e.g., runway latency or punishment breakpoint) versus each individual's mean parental phenotype (Falconer and Mackay, 1996).

Inbred rats, ACI, BN, BUF, FSH, M520, and WKY, were screened on the runway and a subgroup of each strain tested on control tasks in the following order: locomotion, progressive punishment, PR, and shuttle box shock escape. A new group of WKY (*n* = 5) and ACI (*n* = 6) was tested on progressive punishment for cocaine.

c-Fos counts: after behavioral experiments were done, animals were administered 0.75 mg/kg cocaine intravenously and killed 1 h later and brains processed for histology as noted above. Counts were collected from four rostrocaudal RMTg sections (AP = 6.5–7.1) for each animal and averaged for total counts per animal.

### Statistical analysis

All statistical analyses were completed using GraphPad Prism 7.00 and SAS v9.4. We indicate *p* values <0.0001 as *p* < 0.0001 and provide exact *p* values for those >0.0001.

To ensure that the runway latency data met the assumptions of normality, the corresponding data was log-transformed prior to hypothesis testing, which was conducted based on a general linear mixed modeling (GLMM) approach (Kizilkaya and Tempelman, 2005). GLMMs are especially useful for modeling longitudinal data by accounting for within-subject correlation, in this case using random effects for rats. The GLMMs contained fixed effects for generation (i.e., parent, offspring of low/high avoiders f1, offspring of low/high avoiders f2), sex, trial, and a generation by trial interaction term. For analyses of latency data pooled across trials 4–7, Kruskal–Wallis tests followed by a Mann–Whitney *U* test for pairwise comparisons were used. The Wilcoxon matched-pairs signed–rank test was used to compare pairs. Correlations were calculated according to the Spearman method. The level of significance was set at *p* < 0.05. All values were expressed as mean ± SEM.

For the rest of the behavioral data, Student's *t* tests, ANOVA, or mixed linear models were used to assess differences in group means. Main effects analyses from one-way ANOVA or Students *t* tests were followed with Bonferroni or Dunnett's post hoc tests to adjust for multiple comparisons.

## Results

### Individual SD rats show large variation in cocaine's delayed aversive effects

**Figure 1.**
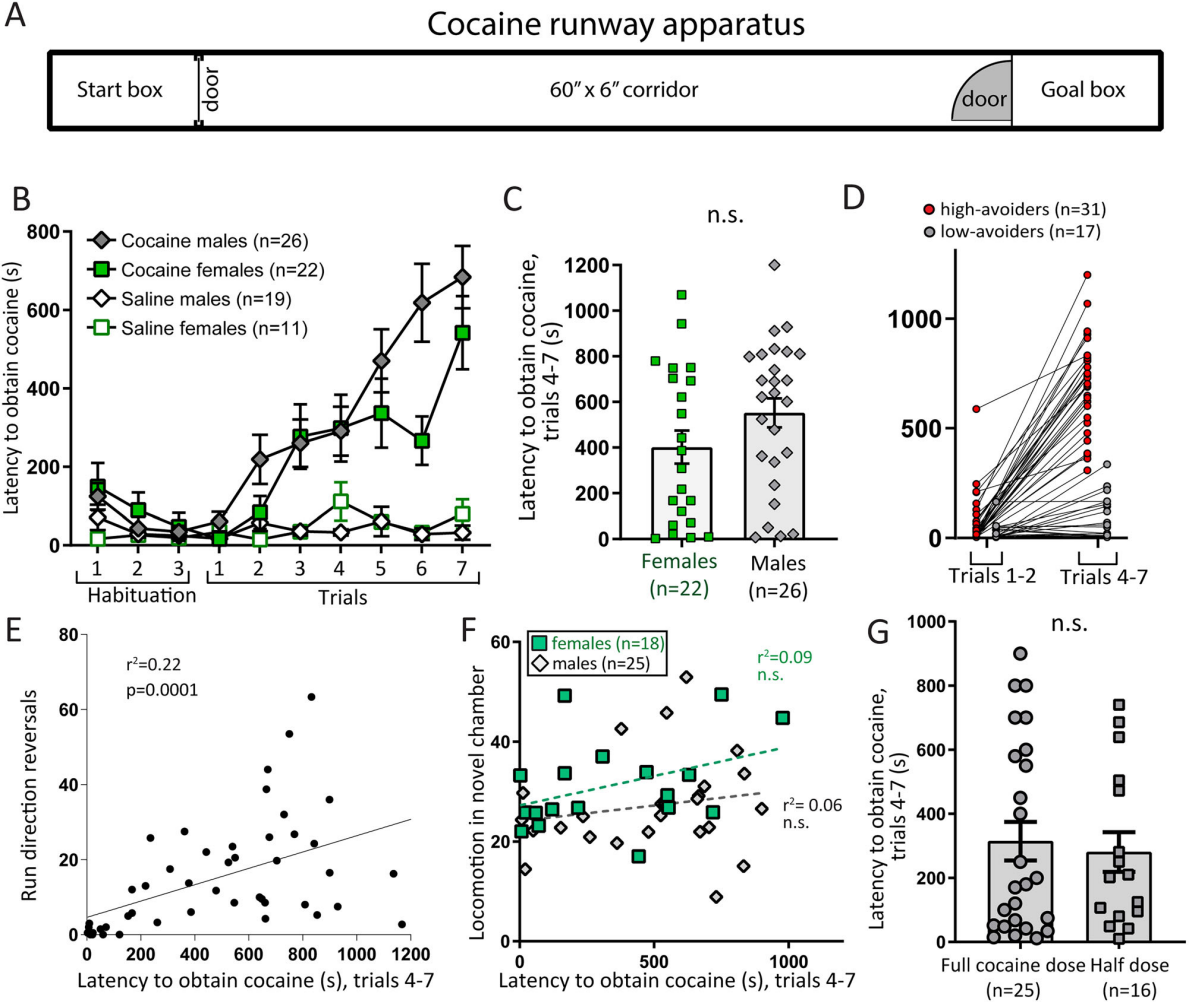
Large variations in cocaine avoidance between individual SD rats in an operant runway task. ***A***, Overhead plan of runway apparatus, with start and goal compartments joined by a 5-ft-long corridor. ***B***, Male and female SD rats traversing the runway to obtain cocaine or saline showed increasing latencies to reach the goal compartment over the course of seven trials in the cocaine condition only (drug × trial interaction, *p* < 0.0001). ***C***, SD rats showed large individual variation in run latencies during Trials 4–7, with no difference between males and females (*n* = 22–26 per group; Mann–Whitney, *p* = 0.14). ***D***, Rats were classified into low- and high-avoider groups by comparing their Trial 4–7 average run latency with a median of 256 s. ***E***, Run latency correlated positively with the number of run direction reversals, consistent with conflicting approach-avoidance to the goal compartment. ***F***, Run latency did not correlate with exploratory locomotion in either males or females. ***G***, Animals tested on a half-size dose (0.375 mg/kg per infusion or ∼0.125 mg per rat) showed similar latencies to obtain cocaine as those receiving the full dose, suggesting that avoidance effects are not due to excessively high doses.*p *<* *0.05*. All data are represented as mean ± standard error of the mean (SEM).

We tested 48 total (26 male and 22 female) SD rats on an operant cocaine-seeking task (Fig. 1*A*), in which animals running to the end of a 5-ft-long (170 cm) corridor received a single cocaine infusion. We also tested 19 male and 11 female control rats that received saline infusions upon reaching the goal compartment.

Consistent with prior reports (Geist and Ettenberg, 1997; Ettenberg et al., 1999; Ettenberg, 2004), we found marked differences between the cocaine and saline groups over the seven trials [i.e., a significant (*p* < 0.0001) drug × trial interaction] but no significant effect of sex (*p* = 0.13; Fig. 1*B*). Post hoc analyses showed that animals running for cocaine had progressive increases in latencies over the course of seven trials, while animals running for saline did not show such increases.

By Trials 4–7, we also saw large individual variations in latencies to obtain cocaine for both males and females (Fig. 1*C*), but with no differences in means between sexes (Mann–Whitney test, *p* = 0.14). We hence subdivided animals into “low-avoiders” or “high-avoiders” based on whether latencies during Trials 4–7 were below or above the median of 256s determined from previous animals run in our lab (Parrilla-Carrero et al., 2021; Fig. 1*D*). In addition to longer run latencies, high-avoiders also had larger numbers of run direction reversals, reflecting alternating approach and retreat behaviors (*n* = 48; *r*^2^ = 0.27; *p* < 0.0001; Fig. 1*E*), consistent with competition between cocaine's dual rewarding and aversive effects reported by others previously (Ettenberg et al., 1999).

For all animals, the runway apparatus was initially novel to the animal, raising the possibility that runway behavior was confounded by novelty-induced locomotion, which also varies between animals and correlates with cocaine-seeking (Stead et al., 2006). We had already attempted to reduce novelty-related effects by habituating all animals to the runway apparatus prior to cocaine testing (see Materials and Methods). To further assess this confound, we next tested animals explicitly on locomotor activity in a second (also initially novel) chamber distinct from the runway apparatus. Locomotion in this new chamber did not correlate with runway latencies (males, *n* = 25; *r*^2^ = 0.03; *p* = 0.40; females, *n* = 22; *r*^2^ = 0.10; *p* = 0.21; Fig. 1*F*), indicating that differences in runway latency were not reflective of differences in exploratory activity.

Notably, the cocaine dose we used (0.75 mg/kg or ∼0.26 mg per infusion for an average 350 g rat) is within the range considered rewarding in self-administration studies (Wee et al., 2007; Garcia-Keller et al., 2019) and is far below levels considered toxic (Smith et al., 1991). Furthermore, a separate cohort tested with a half-size dose (0.375 mg/kg) showed a similar latency distribution as the full dose, with no difference in run mean or standard deviation compared with the full dose (298 ± 94 s vs 344 ± 103 s mean ± SEM for full and half doses; *n* = 16–25 per group; mean, Mann–Whitney, *U* = 197.0; *p* = 0.94; standard deviation, *p* = 0.44; Fig. 1*G*).

### Cocaine avoidance is altered by selective breeding in outbred SD rats

From the 48 SD animals screened above on runway cocaine-seeking, we selected six male-female pairs of animals for selective breeding. Four pairs came from the top quartiles of the distribution of run latencies, while two pairs came from the bottom quartiles. In these pairs, individuals were chosen to meet an additional constraint, such that the mid-parent average of exploratory locomotion scores for each pair was within the middle quartiles of the overall population distribution. This was done to avoid inadvertently selecting for differences in exploratory locomotor behavior. Offspring from all pairings were tested on runway cocaine-seeking, and from these animals, the same criteria were used to select six additional breeder pairs (three low and three high avoider) to produce a second generation of offspring.

**Figure 2.**
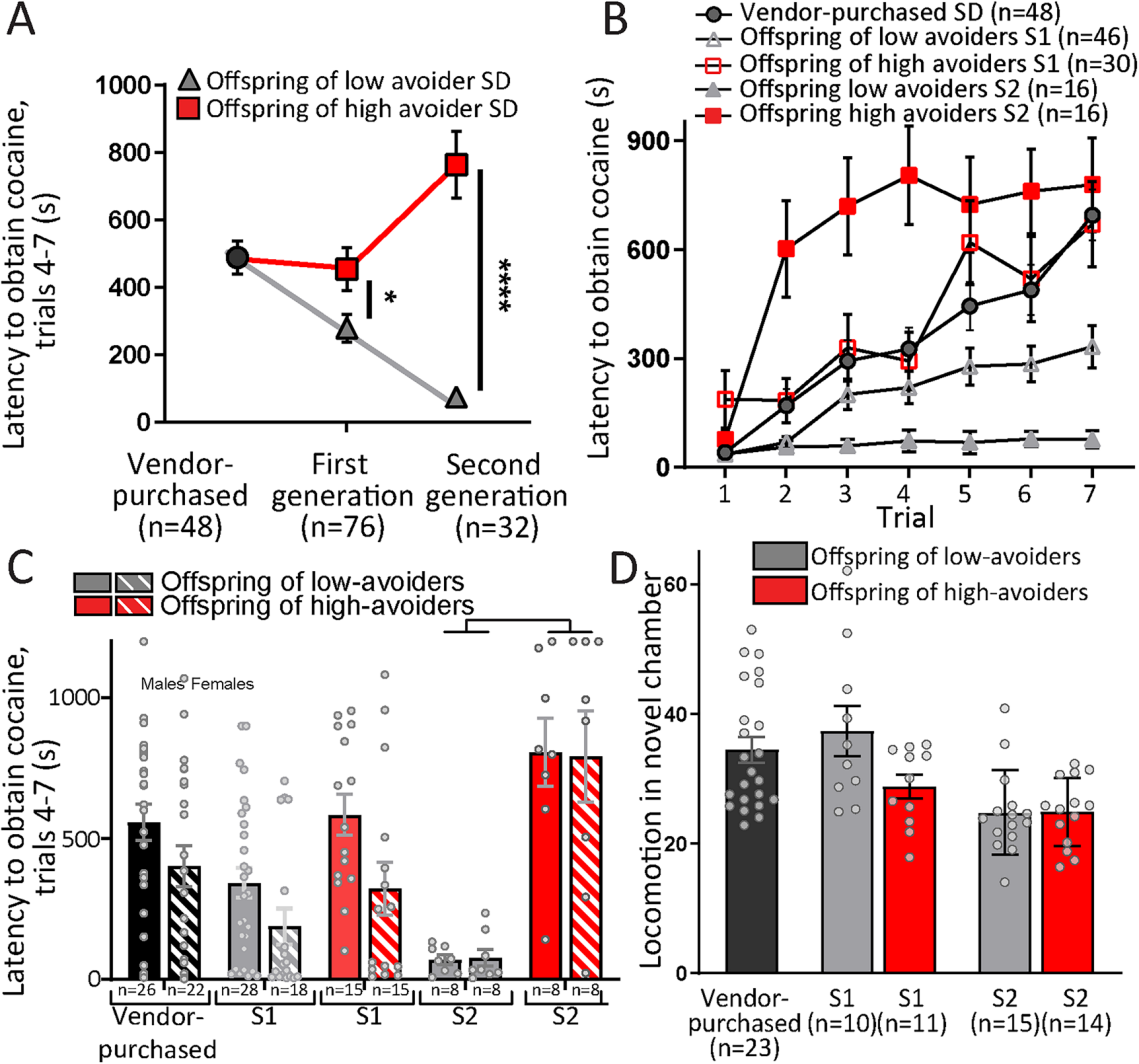
Cocaine avoidance is altered by selective breeding in SD rats. ***A***, Runway latencies to obtain cocaine in SD rats (average of Trials 4–7) were altered after two generations of selective breeding, with offspring of high-avoider parents (red symbols) showing higher latencies than offspring of low-avoider parents (gray symbols). ***B***, Trial-by-trial latencies from offspring of high-avoider parents are higher than offspring of low-avoider parents. By the second generation, high-avoider offspring also manifest these long latencies earlier than other groups. ***C***, Latencies graphed separately by sex and generation. ***D***, Offspring of high- and low-avoider parents did not differ in exploratory locomotion in a novel chamber.

Runway results were initially averaged over Trials 4–7 and tabulated by sex (male vs female), generation (1 vs 2), and parental phenotype (high vs low avoider). A three-way ANOVA showed a main effect of parental phenotype (*f* = 52.81; *p* < 0.0001), and a significant interaction of generation × parental phenotype (*f* = 18.3; *p* < 0.0001), but no overall effect of either generation or sex (*f* = 1.49 and 2.9; *p* = 0.22 and 0.09; Fig. 2*C*). Given the lack of sex effect, males and females were combined for subsequent analyses. We found effects of parental phenotype in both the first and second generations (*p* = 0.04 and *p* < 0.0001, respectively, Mann–Whitney with Bonferroni correction). In both generations, offspring of high-avoider parents showed higher latencies to obtain cocaine than offspring of low-avoider parents, and the magnitude of the difference was particularly large in the second generation, with 12-fold higher run latency in offspring of high- versus low-avoider parents (Fig. 2*A*).

Analysis of trial by trial latencies further showed that second-generation offspring of high avoiders not only showed greater slowing of latencies across trials but also showed faster development of this latency increase, with latencies surpassing 500 s on Trial 2, whereas no other group exceeded 500 s latencies before Trial 5 (GLMM; generation × trial interaction, *p* = 0.001; Fig. 2*B*).

Despite our attempt to avoid altering exploratory locomotion, this measure showed a small reduction from Generation 1 to 2 (two-way ANOVA of generation and parental phenotype, main effect of generation, *F*_(1,46)_ = 14.21; *p* = 0.0005) that was not dependent on parental runway phenotype (*F*_(1,46)_ = 3.71; *p* = 0.06; Fig. 2*D*).

In the first generation, the realized heritability of cocaine runway latency was 0.49 (95% confidence interval 0.32–0.66), as determined by the slope of the offspring–parent regression (Falconer and Mackay, 1996). However, the accuracy of this value is limited by the relatively small number of breeder pairs assessed. Also, the commercial vendor of the SD rats does not provide information about the pedigree of individual animals. Hence, some of the initially purchased animals could have been littermates, potentially reducing available genetic diversity. This would in turn reduce phenotypic differences arising from such diversity. Because of this shortcoming of SD rats, we repeated our selective breeding experiment using NIH HS rats. This strain is an intercross of eight inbred rat strains that are advantageous for fine mapping of genetic loci (Hansen and Spuhler, 1984; Solberg Woods and Palmer, 2019) and for which parentage is well documented.

### Cocaine avoidance is altered by selective breeding in HS rats

**Figure 3.**
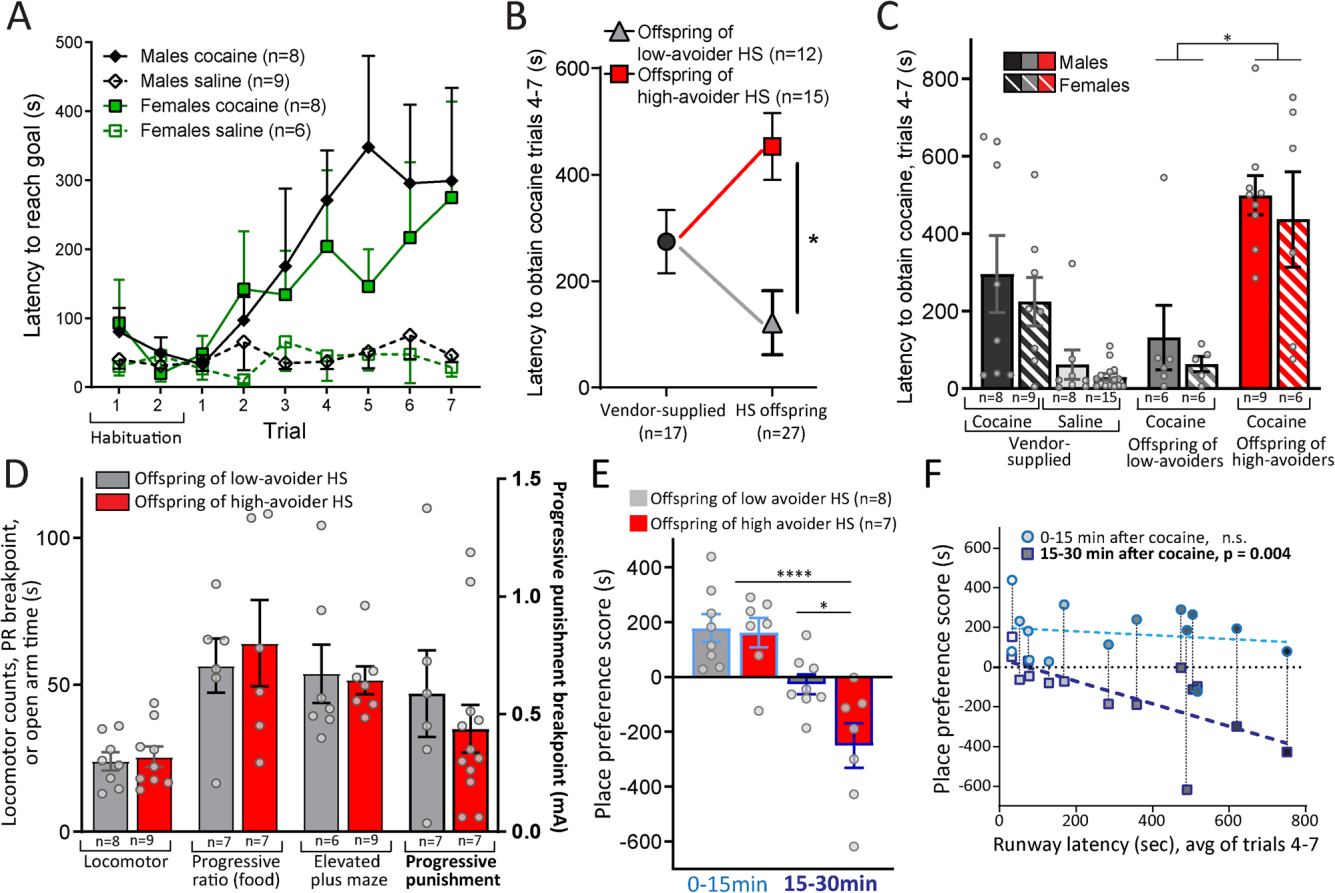
Cocaine avoidance is altered by selective breeding in HS rats. ***A***, HS rats running for cocaine but not saline show progressive increases across seven trials in latency to reach goal compartment. ***B***, Runway latency to obtain cocaine in HS rats was altered by selective breeding after one generation. ***C***, HS males and females did not differ in latency to obtain cocaine. ***D***, Offspring of high and low cocaine avoiders did not differ in PR, punishment resistance, locomotion, or open arm time on an elevated plus maze. ***E***, Cocaine produced strong CPP (light gray bars) in both high- and low-avoiders rats placed into conditioning chambers 0–15 min after intravenous cocaine infusions. However, when placed into chambers 15–30 min postinfusion (dark gray bars), high cocaine avoiders but not low avoiders showed a CPA. ***F***, Runway latencies to obtain cocaine correlated negatively with CPP scores from the 15–30 min delay condition but not 0–15 min condition. *p *<* *0.05*; ****p *<* *0.0001.*

Because HS rats have not previously been tested on the cocaine operant runway task, we first screened 16 HS rats (eight males, eight females) on this task to determine whether HS rats also develop conditioned avoidance to cocaine in a manner similar to SD rats. As controls, we tested an additional 15 HS rats (nine males, six females) running for saline. Breeding records from the strain provider indicated that none of these rats were littermates of each other and indeed did not share any parents in common. We found effects of drug (cocaine vs saline), trial number, and drug × trial interaction, but not sex (three-way ANOVA, cocaine vs saline, *F*_(1,243)_ = 30.23; *p* < 0.0001; sex *F*_(1,243)_ = 1.25; *p* = 0.26; interaction trial × drug *F*_(8,243)_ = 1.99; *p* = 0.049; Fig. 3*A*). Post hoc tests again showed that run latencies for cocaine but not saline increased with trial number, reflecting conditioned avoidance of cocaine but not saline as trials progressed. From the cocaine-tested animals, we selected two male/female pairs each from animals in the highest and lowest quartiles of run latencies and obtained one litter of offspring from each of these four pairs. Offspring of the high-avoider parents had fivefold longer latencies to obtain cocaine than offspring of the low-avoider parents (Mann–Whitney, *U* = 14; *n* = 26; *p* = 0.001; Fig. 3*B*). This difference was independently significant for both males and females, with no sex differences in either parental or offspring groups, and hence we combined sexes for subsequent analyses (two-way ANOVA, *n* = 59 per group; generation, *F*_(2,37)_ = 11.22; *p* = 0.0002; sex, *F*_(1,37)_ = 0.87; *p* = 0.36; interaction, *F*_(2,37)_ = 10.43; *p* = 0.90; Tukey's test for multiple comparisons, male offspring low avoiders vs high avoiders, *n* = 6–9; *p* = 0.0027; female offspring low avoiders vs high avoiders, *n* = 6 per group; *p* = 0.0025; Fig. 3*C*).

As in the SD rats, runway latencies in the HS rats could have been confounded by differences in generalized exploratory behavior, which is itself heritable, and could have been selected for inadvertently. However, this appears unlikely, as offspring of low- versus high-avoider parents did not differ in exploratory locomotion in a novel chamber (Student's *t* test, locomotion, *n* = 8–9 per group, *t*_(15)_ = 0.32). Likewise, offspring of low- versus high-avoider parents did not differ in breakpoints in a PR task (*p* = 0.75; *n* = 6 per group; *t*_(10)_ = 0.44), indicating that we also did n't inadvertently select for differences in generalized motivated behavior (Fig. 3*D*). Lastly, differences in cocaine avoidance could have been due to generalized differences in aversive learning. Hence, we also tested HS offspring on a progressive punishment task (Vento et al., 2017; Li et al., 2019), in which animals pressed a lever for a food reward followed immediately by a mild footshock, whose amplitude increases progressively across trials until animals cease pressing. The highest footshock intensity endured is recorded as a “breakpoint,” indicating the amount of punishment required to inhibit food-seeking. We found no difference between offspring of high and low cocaine avoiders in shock breakpoint (Student's *t* test, *n* = 6 per group; *t*_(10)_ = 0.4404; *p* = 0.67; Fig. 3*D*), indicating that differences in cocaine avoidance were not explained by general differences in aversive learning.

The realized heritability in the HS breeding experiment was 0.63 (95% confidence interval 0.27–0.98), assessed by the slope of the offspring–parent regression.

### Cocaine has dual rewarding and aversive effects in HS rats, with only the aversive component correlating with runway latency to obtain cocaine

Earlier work with SD rats showed that high-avoider rats in the runway task exhibited stronger conditioned place aversion (CPA) to cocaine than low-avoider rats (Ettenberg et al., 2015; Parrilla-Carrero et al., 2021), indicating consistency between two separate measures of cocaine avoidance. Since little is known about cocaine avoidance in HS rats, we tested new groups of male HS rats on the runway task and subdivided them into high and low cocaine avoiders using a median split. We then conducted two separate CPP tests in each rat, using either a “0–15 min” condition, in which rats were confined to conditioning chambers 0–15 min after cocaine infusions or a “15–30 min” condition, in which animals were confined to conditioning chambers 15–30 min after cocaine (0.75 mg/kg, i.v. dose).

In the 0–15 min condition, both high- and low-avoider HS rats showed similar levels of CPP. However, in the 15–30 min delay condition, high-avoider rats showed CPA whereas low-avoiders did not (two-way ANOVA, 0–15 vs 15–30, *F*_(1,26)_ = 29.7; *p* = 0.001; high- vs low-avoiders, *F*_(1,26)_ = 4.504; *p* = 0.04; Bonferroni’s test, *n* = 7–8 per group; high avoiders 0–15 vs 15–30; *p* = 0.04; Fig. 3*E*). The order in which animals were tested on the two conditions (0–15 and 15–30 min) was counterbalanced between animals, with a single “extinction” session in between, during which rats explored all chambers of the apparatus without cocaine. During extinction sessions, rats showed no preference for either side.

Analysis of individual animals further showed that run latencies correlated negatively with place preference scores in the 15–30 min condition, but not in the 0–15 min condition (*n* = 15; *r*^2^ = 0.48; *p* = 0.004; *r*^2^ = 0.028; *p* = 0.5, respectively; Fig. 3*F*), again indicating that slower runway latencies are associated with larger avoidance of the cocaine chamber where animals were placed 15–30 min postdrug.

We did not see systematic biases toward one side or the other of the CPP chamber prior to conditioning. Furthermore, the standard deviation of the baseline preference score was 12.1% of the total session duration, indicating a lack of extreme baseline preferences, as well as ample ability for conditioning to occur in either direction. Finally, the magnitude of baseline preference (black vs white-walled side) did not correlate with runway latency to obtain cocaine (*p* = 0.98; *r*^2^ = 0.00004).

### Cocaine avoidance and punishment resistance behaviors differ between inbred rat strains

As noted above, HS rats are a mixture of eight inbred rat strains, raising the possibility that individual differences in HS rats might have arisen from differences in these constituent strains. Of the eight inbred strains that constitute the HS strain, we tested the six that were still commercially available: the ACI, BN, BUF, FSH, M520, and WKY strains. Two other HS constituent strains (MR and WN) are no longer available from commercial vendors. We also tested LEW inbred rats, which are not one of the HS constituent strains but have been previously studied due to its heightened preference for several types of addictive drugs, including cocaine (Cadoni, 2016). All animals were tested on runway cocaine-seeking, as well as the same control tasks used in the HS rats, namely, punished food-seeking, PR food-seeking, and exploratory locomotion. All inbred runway tests were conducted in male rats, constituting a limitation relative to SD and HS rats in which both males and females were tested.

**Figure 4.**
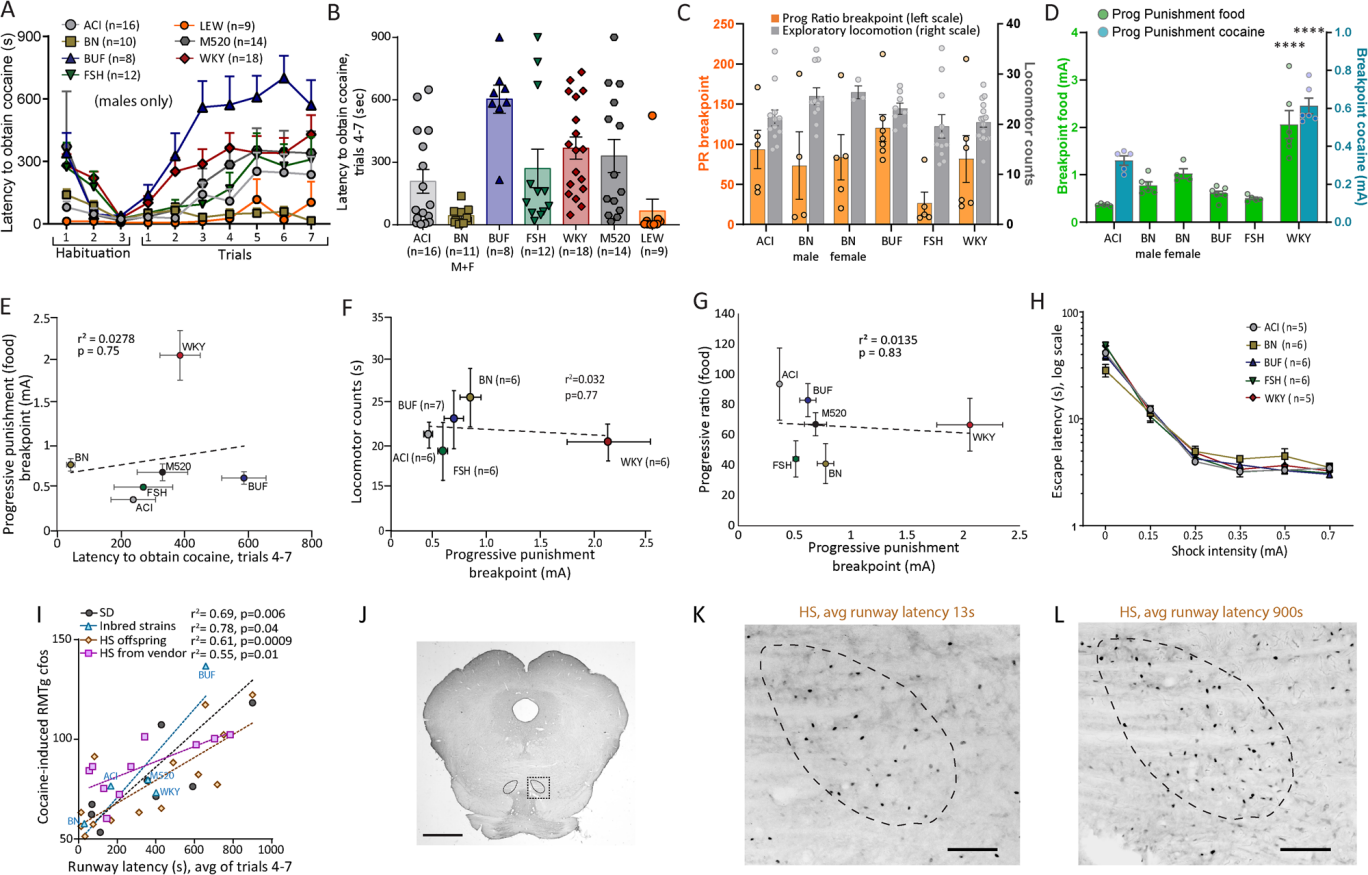
Cocaine-seeking, punishment, PR, and locomotor measures vary independently of each other between strains and individual rats. ***A***, Latency to obtain cocaine in operant runway task differed markedly between six inbred rat strains: ACI, BN, BUF, FSH, LEW, and WKY. Six of these seven strains (all except Lewis) are constituents of the HS strain. ***B***, Bar graph showing same animals as in ***A***, with each symbol showing a single animal's latency averaged over Trials 4–7. ***C***, Inbred strains did not differ from each other in PR food-seeking or exploratory locomotion. BN female and male rats are shown separately, while all other strains were tested in males only. ***D***, WKY showed higher footshock breakpoints than other strains in a progressive punishment food-seeking task and showed higher breakpoints than ACI rats in the same task when using cocaine rewards, reflecting greater punishment resistance for both reinforcers. ***E***, Across strains, punishment resistance (for food rewards) varied independently of cocaine avoidance. ***F***, Across strains, exploratory locomotion in a novel environment varied independently of punishment resistance. ***G***, Across strains, PR breakpoints for food rewards varied independently of punishment resistance. ***H***, Latency to escape acute footshock did not differ between any inbred strains at any shock intensity. ***I***, Cocaine-induced c-Fos in the RMTg correlated with cocaine runway latency in four different groups: vendor-purchased HS rats, vendor-purchased SD rats, offspring of high- and low-avoider HS rats, and inbred rat strains. For HS and SD rats, each symbol represents one animal, whereas for inbred strains (blue triangles) each symbol represents the average value of a single strain. Note that some symbols partly overlap. ***J***, Low-magnification photograph of midbrain sections from which RMTg is sampled (dashed outline indicates region shown at higher magnification in ***K*** and ***L***). Scale bar, 1 mm. ***K***, High-power photographs of c-Fos immunoreactivity in an SD rat exhibiting low runway latency or (***L***) high runway latency, showing greater numbers of c-Fos–positive nuclei in the latter. Scale bars, 100 µm. *p *<* *0.05*; ****p *<* *0.0001.*

On the runway task, we observed consistent differences between strains (Fig. 4*A*; *F*_(5,71)_ = 8.921; *p* < 0.0001 for strain; *F*_(4,300)_ = 13.28; *p* < 0.0001 for session, two-way mixed model with session as a repeated measure). Post hoc tests show that BUF had particularly high latencies, while LEW and BN rats had particularly low run latencies relative to other strains, averaging 586 ± 70, 66 ± 57, and 43 ± 13 s, respectively (Kruskal–Wallis, *H*_(5)_ = 25.15; *p* < 0.0001 Dunnett's test; *n* = 8–12 per group; Fig. 4*B*).

As before, we tested several control tasks to determine whether runway latency differences were explained by general differences in motivated behavior or locomotor exploration. Again, these were largely tested in male rats, along with a small set of female BN rats. No strain differences were seen in either PR or exploratory locomotion (one-way ANOVA, *p* > 0.05 each; Fig. 4*C*, orange and gray bars). We did find differences in punished food-seeking between inbred strains (*p* < 0.0001; *F*_(5,30)_ = 21.38, one-way ANOVA), with WKY rats having higher breakpoints than all other strains (Tukey's post hoc test; Fig. 4*D*, green bars). We next tested two specific strains, WKY and ACI, on the same punishment task but with cocaine rewards and again saw that WKY rats had higher breakpoints than ACI (Student's *t* test, *n* = 6 and 5 for WKY and ACI; *t*_(9)_ = 5.794; *p* = 0.0003; Fig. 4*D*). Interestingly, whereas the higher punishment breakpoint in WKY rats would be consistent with reduced aversive learning, WKY runway latencies were also higher, consistent with increased aversion to cocaine, indicating opposite directions of differences in aversive processing of cocaine and shock stimuli. Overall, shock breakpoints in the progressive punishment task did not correlate with cocaine avoidance, either between strains or between individual rats (*n* = 6–7 per group; *r*^2^ = 0.031; *p* = 0.78 between strains; *n* = 33; *r*^2^ = 0.04; *p* = 0.75 between individuals; Fig. 4*E*, showing correlation by strain). Furthermore, across all five strains, PR breakpoints and progressive punishment shock breakpoints did not correlate with each other (*n* = 6 per group; *r*^2^ = 0.013; *p* = 0.86; Fig. 4*G*). Differences in shock breakpoints also did not correlate with exploratory locomotion between individuals or across strains (*n* = 33; *r*^2^ = 0.04; *p* = 0.74; *n* = 6–7 per group; *r*^2^ = 0.032; *p* = 0.77, respectively; Fig. 4*F*, showing correlation across strain only).

We performed one final control task to measure of latency to escape acute footshocks (0.15–0.7 mA) in a two-way shuttlebox. We found no strain differences at any shock intensity, suggesting that differences in cocaine avoidance (and also punishment sensitivity) were again not due to generalized differences in aversive processes (repeated measures two-way ANOVA, inbred strain, *F*_(4,22)_ = 4.403; *p* = 0.0091; shock intensity, *F*_(5,110)_ = 687; *p* < 0.0001; interaction, *F*_(20,110)_ = 7.777; *p* < 0.0001; Bonferroni’s test, *n* = 5–6 per group; Fig. 4*H*).

### Cocaine-induced c-Fos in RMTg correlates with runway latency across multiple strains

Prior work in SD rats showed that single cocaine doses increase expression of the immediate early gene product c-Fos in the RMTg to a greater degree in high versus low avoiders of cocaine (Jhou et al., 2013; Parrilla-Carrero et al., 2021; Chao et al., 2023). We examined whether this result would replicate in our current cohort and extend to HS rats and found that indeed, RMTg c-Fos correlated positively with run latencies in vendor-purchased HS rats, vendor-purchased SD rats, and selectively bred HS rat offspring (*n* = 5–16 per group; *p* < 0.05 each; Fig. 4*I*–*L*).

## Discussion

We confirmed that individual SD rats differed markedly in the degree to which they exhibit avoidance responses to cocaine, consistent with prior work (Parrilla-Carrero et al., 2021; Chao et al., 2023). We then showed that rats from the HS strain, which has increasingly been used in heritability studies (Hansen and Spuhler, 1984; Solberg Woods et al., 2010; Wagner et al., 2023), exhibited similarly high levels of individual variability in both runway and CPA measures of cocaine avoidance and that these two behavioral measures of avoidance correlated with each other in the HS strain, as had been observed earlier in SD rats. We further demonstrated that runway cocaine latency was markedly altered after one or two generations of selective breeding in either SD or HS rats, consistent with a heritable influence that could be either genetic or nongenetic. Results of several control tasks showed that cocaine runway latency differences were not explained by generalized differences in exploratory locomotion or aversive learning. Notably, one of these control tasks, a test of punished reward-seeking, is itself implicated in addiction-like behaviors, as one hallmark of addiction is the pursuit of rewards despite increasingly severe negative consequences. Lastly, runway cocaine-seeking, along with punished reward-seeking, also differed between several inbred rat strains and did so independently of each of other, suggesting both are influenced by distinct heritable factors.

A number of limitations of this study should be noted. While the HS individuals we obtained from the strain provider did not share any common parents, the vendor-purchased SD rats are of unknown parentage, and we cannot rule out that many of them could have been littermates. Such relatedness would reduce the ability to detect genetic heritable influences, as there would be fewer genetic variations to select for (or against) in the breeding process, and hence true heritability could be higher than what is calculated here. Overall, the number of breeder pairs in both experiments was low, and hence heritability estimates for either strain are prone to influence by chance effects. Furthermore, the current study cannot distinguish between genetic and nongenetic influences on behavior. For example, differences in maternal behavior can influence offspring behavior via nongenetic means (Wolf and Wade, 2009), but we did not perform cross-fostering to control for such effects. Lastly, several caveats apply to the cocaine runway task, which could in principle have been confounded by differences in motoric performance or generalized motivation. However, we found that runway latencies for cocaine did not correlate with differences in exploratory locomotion, PR food-seeking, nor shock-punished food-seeking. In contrast, cocaine runway latencies did correlate with CPA in HS rats in the current study, consistent with a similar correlation observed in SD rats in prior work, strongly suggesting that runway latencies are indeed related to aversive effects of cocaine. However, although this corroboration was performed for HS rats in the current study, inbred rat strains were only tested on the runway, and not CPA.

Interestingly, one of our control tasks, punished food-seeking, may also model some aspects of addiction-related behaviors in its own right. In human populations, some drug users readily curtail intake in the face of rising external costs, e.g., financial or social, whereas other individuals have much more difficulty doing so (Lopez-Quintero et al., 2011). Because prior work showed that cocaine aversion and punishment learning performance both strongly depend on RMTg function (Jhou et al., 2009a,b; Vento et al., 2017; Li et al., 2019), we had initially expected that runway and punishment behaviors would vary together. However, this was not the case, as strain differences in punishment task performance were independent of differences in cocaine runway performance. This suggests that different underlying mechanisms are influencing these aspects of drug-seeking, even though they both involve aversive learning and share some convergent neural circuit substrates.

We chose a runway operant task as our main measure of operant cocaine-seeking rather than the more commonly used lever press self-administration model, as it is particularly strongly influenced by aversive effects of cocaine. However, the two types of tasks do appear related in some aspects. Specifically, when runway high-avoiders” were subsequently tested on lever press cocaine self-administration, they exhibited reduced lever pressing for cocaine during the first three sessions of lever press training, relative to low avoiders (Chao et al., 2023). This result suggests that cocaine's aversive effects might slow the initial acquisition of drug-seeking. However, as training continued, the runway high avoiders eventually increased lever pressing to match the low avoiders, suggesting that the initial influence of cocaine's aversive effects can be overcome with additional training. Interestingly, after subsequent extinction training, followed by reinstatement tests, high avoiders showed reduced cue-induced reinstatement of lever pressing, relative to low avoiders (Chao et al., 2023), suggesting that runway high avoiders continue to be “protected” against drug-seeking even at this much later stage of testing.

Our findings are broadly consistent with decades of studies showing that addiction-related behaviors are influenced by heritable factors in both humans and rodents (Holtz and Carroll, 2016; Deak and Johnson, 2021). It is unknown to what degree findings in rodents versus humans apply to each other, but some genome-wide association studies (GWAS) in rodents have notably identified similar genes and/or gene networks as implicated in human populations (Huggett et al., 2021; Wright et al., 2023). Such similarities raise the possibility that animal and human studies could be used to complement each other. For example, in human populations, heritable factors are thought to explain ∼40–60% of phenotypic variance in addiction (Deak and Johnson, 2021; Gerring et al., 2024), but the gene–phenotype relationship appears extremely complex, and specific loci identified by GWAS studies involving large numbers of human subjects have so far explained only a small portion of the overall heritable influence (Zhou et al., 2020; Hatoum et al., 2023). Animal models, despite their limited ability to mimic the full complexity of human addiction, can allow much more control over behavioral paradigms, environmental factors, and population genetic makeup. Hence, GWAS studies in animals, including SD and HS rats (Solberg Woods and Palmer, 2019; Gileta et al., 2022), may require much smaller populations to identify comparable numbers of loci (Wright et al., 2023) and can produce findings more amenable to subsequent mechanistic studies (Lara et al., 2024; King et al., 2025; Kuhn et al., 2025). Whether such an approach would help elucidate genetic contributions to the cocaine avoidance or punishment learning tasks studied here has yet to be determined but may be a promising area for additional study.

## Data Availability

All raw data will be made available on request.

## Synthesis

Reviewing Editor: Tod E Kippin, University of California Santa Barbara Decisions are customarily a result of the Reviewing Editor and the peer reviewers coming together and discussing their recommendations until a consensus is reached. When revisions are invited, a fact-based synthesis statement explaining their decision and outlining what is needed to prepare a revision will be listed below. The following reviewer(s) agreed to reveal their identity: Malia Bautista.

This resubmitted manuscript provides interesting evidence that behavioral responding to cocaine's aversive properties is heritable, as selectively breeding for different avoidance phenotypes strengthens avoidant or non-avoidant behaviors in Sprague Dawley and HS stock strains. The authors further explore this effect across a variety of rat strains, characterizing differences in avoidance behavior for cocaine across both inbred and outbred strains, with minimal effects on baseline exploratory locomotion and progressive ratio breakpoints. A vast majority of the previous reviewers' comments were addressed, including the general de-emphasis of clinical phenomena. However, given the significant revisions from the prior version, several issues are noted with the current version of the manuscript that need to be addressed.

1. The authors have included rationale for excluded subjects but have not provided exclusion criteria for "failure to show exploratory behavior on the runway apparatus." However, for experimental rigor, the maximal amount of "failed" habituation sessions before exclusion should be included.

2. How was heritability calculated? This needs to be clarified and stated in the methods in addition to the results text.

3. Across the entirety of the figures, there are inconsistencies in whether the individual points were included. To be transparent on variability within datasets, please provide the individual points on all bar graphs.

4. Combining PR breakpoint, locomotion, and punishment breakpoint into a singular graph (Figure 4C) makes the graph confusing. If the purpose of the graph is to highlight the main effect in progressive punishment breakpoint, what is the rationale for combining this data with PR breakpoint for food and locomotion? What is the rationale for separating the BN males and females in this graph but not the others? What are the reported sample sizes for these groups?

5. It is misleading to extrapolate the progressive punishment data ACI and WKY strains to conduct a t-test (Figure 4D). What additional information does this graph provide that 4C does not?

6. Interesting c-fos data in figure 4I. Subject numbers and representative images from low and high avoiders would provide compelling visual evidence of this correlation. Moreover, inbred strains have a low subject number, n=3, with no indication which specific inbred strains the data were derived.

7. The discussion focuses on a few GWAS studies to demonstrate the relative heritability of substance use disorders. It is important to address previous GWAS studies that have been done on specific rat strains. Thus, discussion of currently available GWAS data on SD rats (PMID: 35639796) would be beneficial to place this manuscript within the context of the heritability field.

8. The titles "Independent heritability of aversive learning" "runway operant cocaine-seeking" (Line 111) are misleading.

Given that the animals received the cocaine infusion regardless of their behavior, "operant" is not an accurate term as the animals' behavior did not alter the outcome of the task. Similarly, "learning" seems like an overreaching statement of the runway task. Rather than learning, this task more accurately represents the decision making in response to cocaine's reinforcing and aversive effects.

## Author Response

1. The authors have included rationale for excluded subjects but have not provided exclusion criteria for "failure to show exploratory behavior on the runway apparatus." However, for experimental rigor, the maximal amount of "failed" habituation sessions before exclusion should be included.

We now clarify that animals that "failed" habituation is defined by failure to enter the goal box within 60 seconds of opening the start door, and that animals were excluded after four consecutive habituation trials.

2. How was heritability calculated? This needs to be clarified and stated in the methods in addition to the results text.

The method of calculation is now stated in the methods as the slope of the regression line fit to a scatterplot of offspring phenotype vs parental mean phenotypes.

3. Across the entirety of the figures, there are inconsistencies in whether the individual points were included. To be transparent on variability within datasets, please provide the individual points on all bar graphs.

Individual datapoints are now added for all bar graphs.

4. Combining PR breakpoint, locomotion, and punishment breakpoint into a singular graph (Figure 4C) makes the graph confusing. If the purpose of the graph is to highlight the main effect in progressive punishment breakpoint, what is the rationale for combining this data with PR breakpoint for food and locomotion? What is the rationale for separating the BN males and females in this graph but not the others? What are the reported sample sizes for these groups? We agree the progressive punishment data makes more sense in a separate graph, and it is now moved to 4D. Regarding the females and males being separated in this graph, indeed there are some graphs where sex is not specified. In those cases, behavior was tested in males only, and that is now clarified.

5. It is misleading to extrapolate the progressive punishment data ACI and WKY strains to conduct a t-test (Figure 4D). What additional information does this graph provide that 4C does not? We apologies for the confusion here. Fig. 4C represents sessions with food rewards, while 4D shows sessions with cocaine reward. These are two different tests, which is now clarified by placing the two experiments on the same graph, using green vs blue bars (revised Fig. 4D), so it is more obvious that they are different tests with different (albeit complementary) outcomes.

6. Interesting c-fos data in figure 4I. Subject numbers and representative images from low and high avoiders would provide compelling visual evidence of this correlation. Moreover, inbred strains have a low subject number, n=3, with no indication which specific inbred strains the data were derived.

We now clarify that the n for the inbred strains is the number of STRAINS, n=5, with each data point representing an average of 6-8 animals. The strain identities are now placed next to the data points to make this more clear, and also to identify datapoints that are partially obscured by other symbols. Also, two representative images of cFos from HS rats are now shown.

7. The discussion focuses on a few GWAS studies to demonstrate the relative heritability of substance use disorders. It is important to address previous GWAS studies that have been done on specific rat strains. Thus, discussion of currently available GWAS data on SD rats (PMID: 35639796) would be beneficial to place this manuscript within the context of the heritability field.

We agree this is a useful reference, as it uses SD rats, unlike most other GWAS studies in rodents that have used HS rats. It is now added to the discussion with a particular note about the strain used.

8. The titles "Independent heritability of aversive learning" "runway operant cocaine-seeking" (Line 111) are misleading. Given that the animals received the cocaine infusion regardless of their behavior, "operant" is not an accurate term as the animals' behavior did not alter the outcome of the task. Similarly, "learning" seems like an overreaching statement of the runway task. Rather than learning, this task more accurately represents the decision making in response to cocaine's reinforcing and aversive effects.

Indeed, animals receive cocaine regardless of whether they enter the goal compartment or not. However, animals that run directly toward the goal compartment (rather than waiting for the timeout) on average receive the cocaine much earlier. A considerable literature on delay discounting has shown that time delays are extremely critical for learning. Rewards (and punishers) delivered immediately, or with shorter delays, are more reinforcing than delayed rewards. Hence, animals are ultimately motivated to perform actions that reduce the latency to obtain rewards, while increasing the latency to obtain punishers.

We are puzzled by the reviewer's assertion that runway behavior does not represent learning. We and others have shown repeatedly that the emergence of long latencies this task takes several sessions to develop, and does not occur to saline. In other words, it requires prior experience with behavior-outcome contingencies. Ettenberg and colleagues have shown the same thing, and concluded that this is evidence of a conditioned (i.e. learned) avoidance response. The reviewer suggests that the task instead represents "decision making in response to cocaine's reinforcing and aversive effects". This sounds like a rephrasing of the classic definition operant learning, by which an outcome on one trial alters the behavior of subsequent trials.
